# Comparison of Thin-Prep and cell block preparation for the evaluation of Thyroid epithelial lesions on fine needle aspiration biopsy

**DOI:** 10.1186/1742-6413-5-3

**Published:** 2008-03-25

**Authors:** Husain A Saleh, Jamal Hammoud, Richard Zakaria, Aurang Zeb Khan

**Affiliations:** 1 Department of Pathology, Wayne State University/Sinai- Grace Hospital, 6071 west Outer Drive, Detroit, MI, 48302, USA; 2 Division of Endocrinology, Genesys Regional Medical Center, One Genesys Parkway, Grand Blanc, Michigan, 48439, USA; 3 Detroit BioMedical Laboratories, 23955 Freeway Park Drive, Farmington Hills, Michigan, 48024, USA

## Abstract

**Background:**

The objective of this study was to compare the utility of Thin-Prep (TP) cytologic preparation with that of Cell Block (CB) preparation in the diagnosis of thyroid lesions, mainly follicular epithelial lesions, by fine needle aspiration biopsy (FNAB). Feasibility of using the TP slides for immunocytochemical stains is also discussed.

**Methods:**

A total of 126 consecutive cases of thyroid FNAB with TP slides and 128 consecutive cases of thyroid FNAB with CB slides were reviewed blindly by two cytopathologists. The presence of colloid, follicular cells, macrophages and lymphocytes/plasma cells were recorded and scored 0–4 on each case based on TP or CB slide review. The cytologic diagnoses were grouped as follows: cyst, colloid nodule, colloid nodule with cystic change, chronic thyroiditis, atypical/neoplastic and non-diagnostic.

**Results:**

The TP slides had higher diagnostic rate than CB slides. The diagnostic yield was 68% of the TP slides whereas only 24% of the CB slides were diagnostic. Also, only 4 atypical/neoplastic lesions were diagnosed on the TP slides and the corresponding direct smears, while 5 cases of atypical/neoplastic lesions were diagnosed on the smears but could not be diagnosed on the corresponding CB slides. Additionally, the TP slides revealed cytologic features that were not observed on the direct traditional smears of the same case.

**Conclusion:**

In thyroid FNAB cases, TP slide preparation is superior to CB slide preparation and is more likely to have greater cellularity for diagnosis and detect atypical/neoplastic thyroid lesions, particularly those of follicular cell origin. Furthermore, TP slides appear to detect helpful diagnostic cytologic features and should be considered complementary to, rather than replacing, direct smears.

## Background

FNAB is a cost effective and perhaps the best procedure for the initial evaluation and diagnosis of thyroid lesions [1,2]. It is simple, safe and reliable especially when performed under ultrasound guidance. Traditionally, cell block preparations were made from the aspirated material rinsed in cytology preservative solution in addition to the direct conventional smears. However, there have been concerns about the effectiveness of the CB method with regards to cellular adequacy and accuracy of diagnosis [3-5]. During the last several years, there has been a surge in liquid-based cytology slide preparation such as Thin-Prep monolayer slide method in non-gyn cytology specimens [6-10] including thyroid FNAB [8-12]. This method is convenient to cytopathologists interpreting theses aspirates since it yields optimal cellularity for evaluation, and studies have shown similar or even better diagnostic accuracy as compared to direct smear method [13-15]. Today, TP slide preparation from the aspirated specimens rinsed in cytofixative solution has gained wide acceptance and is probably more commonly used in thyroid FNAB than CB preparation [3-5,16,17].

A major advantage of the CB method is the ability to perform multiple immunocytochemical stains or other special stains if needed [18]. However, there have been recent studies demonstrating the ability to successfully perform immunocytochemical stains on additional unstained TP slides in thyroid FNAB specimens ([10,19], and [20]), but the results obtained with this approach have not been directly compared with same specimens processed by traditional CB method. In our laboratory, we converted from CB to TP slide preparation of the rinsed aspiration material on thyroid FNA aspirates in early 2007. In this study we analyze our experience with the TP method and compare it to the previously used CB preparation with regards to adequate cellularity and accurate diagnosis.

## Materials and methods

We retrieved the slides and cytology reports of the first 126 cases of thyroid FNA biopsies since converting to the TP method, and also the last 128 cases of thyroid FNA biopsies made with CB method. These FNAB cases were all office procedures done by experienced endocrinologist, with or without ultrasound guidance, usually using 22 or 23 gauge needles connected to a 10 c.c. syringe. The direct smears were mostly alcohol fixed (spray fixation) and stained with Papanicolaou stain. In few cases, half of the smears were air-dried and stained with Diff-Quik stained. The remaining aspirate specimen was rinsed in Saccomono's fluid for CB preparation. After converting to the TP method, the remaining aspirate material was rinsed in the physician's office in buffered fixative, Cytolyt Solution (Cytyc Corporation, Boxborough, MA, USA). In the lab, the specimen was spun down, resuspended in a buffer PreservCyt (Cytyc Corporation) and then processed in Thin Prep Processor 2000 (Cytyc Corporation). The TP, CB and direct smear slides were evaluated blindly by two independent cytopathologists without knowledge of the original diagnosis. The examined slides were scored 0–4 on specific cytologic features as follows: cellularity (0: no cells, 1: rare cells, 2: 8–12 groups of cells, 3: 12–24 groups of cells, and 4: very cellular specimen); colloid (0: no colloid, 1: scant, 2: mild, 3: moderate, and 4: abundant colloid); macrophages (0: no macrophages, 1: rare, 2: mild 3: moderate, 4: many); and lymphocytes/plasma cells (0: no lymphocytes/plasma cells, 1: rare, 2: mild, 3: moderate, and 4: many). The individual value of each cytologic feature was entered in a designed grid immediately after microscopic examination. The cytologic findings of both pathologists were compared and had high interobserver agreement. In few cases, there were minor differences in scoring, the slides of these cases were jointly reviewed and diagnostic concurrence was obtained. The final cytologic diagnoses for both the TP and CB on each case were classified into the following categories according to previously published cytologic criteria: 1. cyst, 2. colloid nodule, 3. colloid nodule with cystic change, 4. chronic thyroiditis, 5. atypical, 6. neoplastic and 7. nondiagnostic.

## Results

Table 1 shows a summary of the total number of each diagnostic category obtained on both the TP and CB slides. There were 86/126 (68%) TP slides diagnostic of various thyroid lesions compared to only 31/128 (24%) of CB slides (table 1). Both preparations had almost the same rate of diagnosis for thyroid cyst with 17/128 (13%) for CB and 16/126 (13%) for TP slides. However, the number of macrophages and the amount of cyst fluid were higher on the TP slides.

**Table 1:** Comparison between the Thin Prep diagnoses and cell block diagnoses of Thyroid FNA's

	**Cyst**	**CN or CN/C**	**Chronic Thyroiditis**	**Atypical**	**Neoplastic**	**Non Dx**	**Total**
Thin Prep	16 (13%)	52 (41%)	14 (11%)	3	1 PTC	40 (32%)	126
Smear	10	51	16	3	1 PTC	45	126
	**Cyst**	**CN/C**	**Chronic Thyroiditis**	**Atypical**	**Neoplastic**	**Non Dx**	**Total**
CB	17 (13%)	10 (8%)	4 (3%)	0	0	97 (76%)	128
Smear	3	63	18	3	2 FN	39	128

FNA: fine needle aspiration, CN: colloid nodule, CN/C: colloid nodule with cystic change, NON-DX: non-diagnostic, CB: cell block, PTC: papillary thyroid carcinoma, FN: follicular neoplasm.

**Figure 1:**
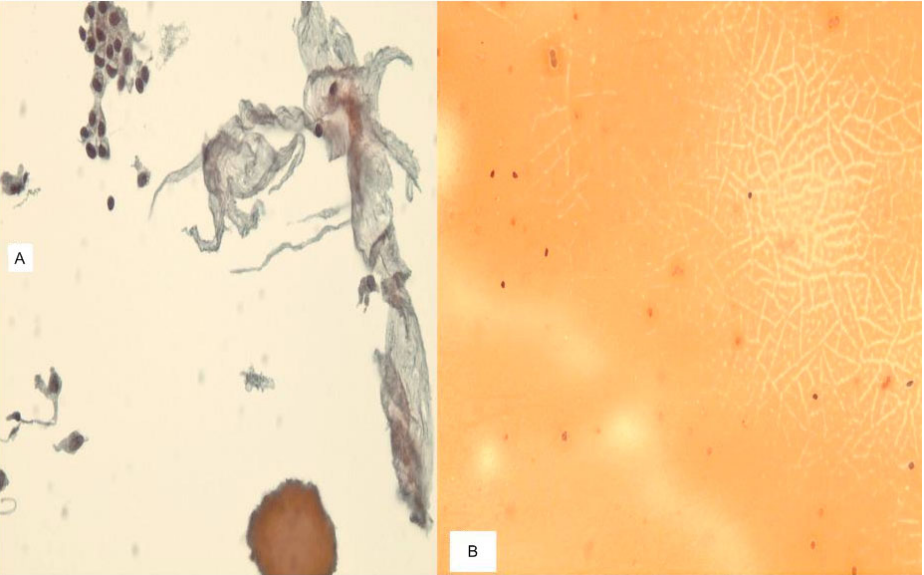
**a. **A TP slide of colloid nodule of thyroid FNA showing a group of bland follicular cells, thick globule of colloid (cast) and thin colloid resembling wrinkled "paper tissue-like material". **b**. Traditional smear of the same case showing typical colloid but no follicular cells (600×, Papanicolaou stain).

**Figure 2:**
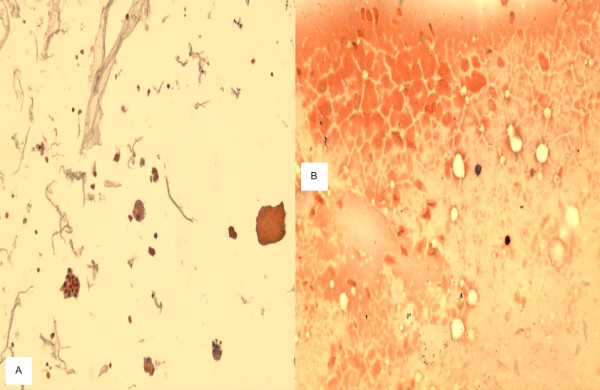
**a**. A TP slide of colloid nodule with cystic change of thyroid FNA showing colloid cast, group of follicular cells and macrophages. **b**. Traditional smear of the same case displaying abundant colloid and macrophages, but no follicular cells (600×, Papanicolaou stain).

The TP slides were more likely to detect colloid nodules and chronic thyroiditis. There were 52/126 (41%) TP slides diagnostic of colloid nodules/colloid nodules with cystic change compared to only 10/128 (8%) diagnosed on CB slides. Most of the cases had more follicular cell groups on TP slides than on the direct smears of the same case (Figs. 1 and 2). Also, TP slides tended to be more cellular than CB slides in cases diagnosed as colloid nodules or colloid nodules with cystic change.

**Figure 3:**
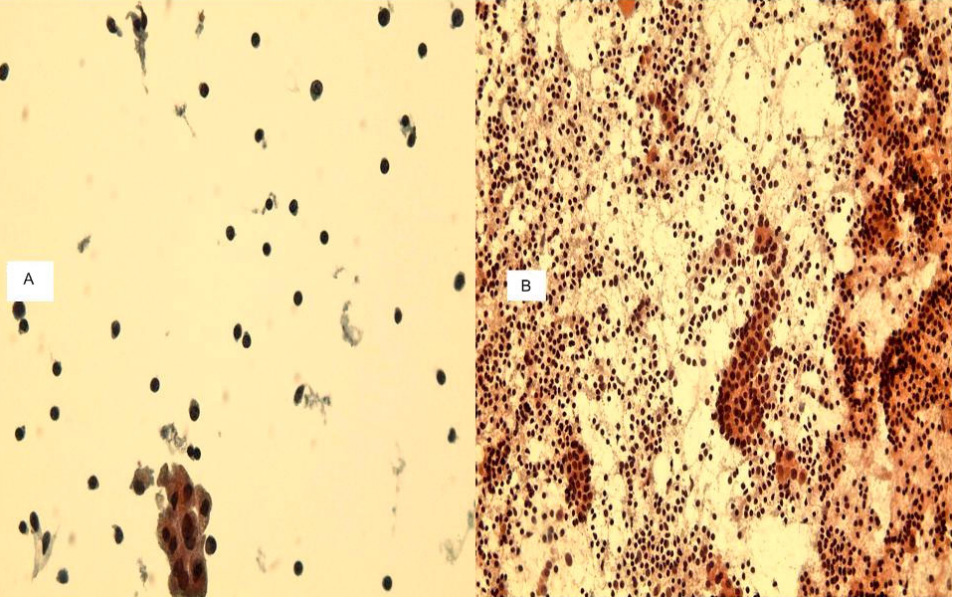
**a**. A TP slide of chronic thyroiditis of thyroid FNA showing many lymphocytes and scattered plasma cells, group of follicular cells and no colloid. **b. **The traditional smear of the same case displaying many lymphocytes, plasma cells and groups of reactive follicular cells. No colloid. (400×, Papanicolaou stain).

Our analysis showed that 14/126 (11%) of TP slides displayed cytologic features consistent with chronic thyroiditis, in contrast to only 4/128 (3%) of CB slides (Fig. 3). Interestingly, TP slides had higher number of lymphocytes and plasmacytes in these cases, and also contained few lymphoid tissue fragments in addition to the singly dispersed lymphocytes and plasma cells. However, cytopathologists should be aware of the presence of mild number of lymphocytes on the TP slides from the lysed blood, and avoid overdiagnosis of chronic thyroiditis.

**Figure 4:**
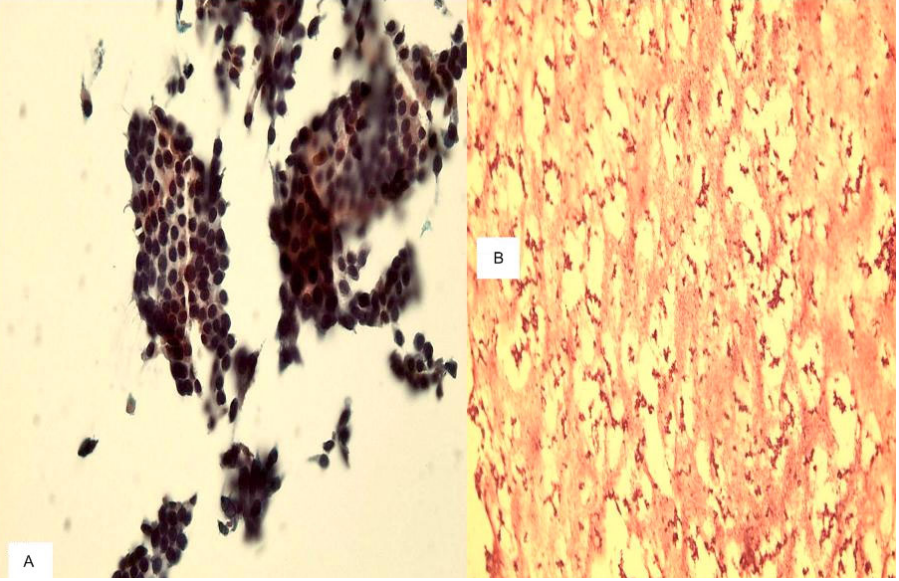
**a. **A TP slide of follicular neoplasm on thyroid FNA showing cellular specimen and groups and 3-dimentional clusters of follicular cells with hyperchromatic nuclei. **b. **The cell block section of the same case shows acellular specimen (400×, Papanicolaou and H&E stains).

More importantly, in the group of FNAB with TP slides, 4 atypical/neoplastic lesions, including one papillary carcinoma, were detected which were also seen on the traditional smears. In contrast, 5 atypical/neoplastic lesions, including two follicular neoplasms (Fig. 4), were identified on traditional smears but were not seen on the corresponding CB slides.

## Discussion

FNAB of the thyroid is a very common procedure for the initial evaluation and diagnosis of thyroid lesions. It is a simple, safe, reliable and cost effective procedure as a first line technique for the diagnostic assessment and management of thyroid lesions. It is also a very popular out-patient clinical procedure that can be performed with or without ultrasound assistance depending on the size of the lesion [1,2]. Usually, an average of three passes is performed and direct smears are made at the time of the procedure. Some of the smears are air-dried and stained with Diff-Quik stained, and others are alcohol-fixed (spray fixed) and stained with Papanicolaou stained. In addition, the remaining aspirate material from each pass is rinsed and flushed in cell preservative solution, such as Cytolyt Solution or Saccomano's fluid, or in saline. Traditionally, CB has been made from this rinsed material and Hematoxylin & Eosin stained slides are prepared in addition to the direct smears, as is done on FNAB of many other organs, like the lung, liver and pancreas. However, during the last decade liquid-based monolayer slide preparation such as the TP method has gained widespread acceptance in the non-gyn cytology practice after a tremendous success in the gyn Pap test cytology [6-11]. As a result, many cytology labs have converted to TP slides preparation from the rinsed aspirate instead of the usual CB preparation. Some laboratories have even elected to make only TP slides without conventional smears or CB slides, especially with thyroid FNAB specimens obtained in physician offices.

There have been several studies comparing the utility and advantages of TP method and the conventional direct smear method in thyroid FNAB samples [12,14,21,22]. Fewer studies, however, were conducted to compare the utility and advantages of TP slide method with that of CB slides in thyroid FNAB specimens [3-5]. A study by Selvaggi and Sanchez found that the contribution of the CBs in the diagnosis of thyroid lesions is minimal because of low cellularity [3]. They also found that the CBs were not helpful in the majority of cases and were contributory in only 31% of all 82 cases that had CB preparation. More importantly, CBs were contributory in only 5 of 23 (21.7%) of neoplastic thyroid lesions. Another study by Siddiqui et. al. of 197 thyroid FNAB cases found that the majority (57.9%) of cases were diagnosed only on conventional smears, either Diff-Quik or Papanicolaou stained [4]. The authors also found that CB slides were diagnostic only in 6.1% of the cases.

In our study, we found that TP slides are much more likely to be diagnostic of colloid nodules, colloid nodules with cyst, chronic thyroiditis and atypical/neoplastic lesions than CB slides. Overall, TP slides were diagnostic of various thyroid lesions in 86/126 (68%) of the cases, while CB slides were diagnostic in only 31/128 (24%) of the cases. The CB and TP slides had similar diagnostic yield only in cases of degenerative thyroid cysts, 17/128 (13%) vs. 16/126 (13%), respectively. In cases of colloid nodules or colloid nodules with cystic change, CB had diagnostic yield of 10/128 (8%) compared to 52/126 (41%) on TP slides. We also found that CB slides were diagnostic of chronic thyroiditis in only 4/128 (3%), while TP slides were diagnostic in 14/126 (11%). More significantly, we found that 5 CB slides failed to identify atypical/neoplastic lesions, including 2 follicular neoplasms that were diagnosed on the corresponding smears. On the other hand, 4 TP slides detected atypical/neoplastic lesions, including 1 papillary carcinoma, that were also diagnosed on the corresponding smears. This sharp contrast is perhaps the most important finding in our study showing that TP slides are more likely to detect atypical/neoplastic lesions than CB slides in thyroid FNAB cytologic evaluation.

Our results are essentially in agreement with those of previous studies which demonstrated good diagnostic yield of TP slides of thyroid aspirates. Frost et al found that TP slides of thyroid aspirates have an 85% diagnostic accuracy, and that preparation of only two TP slides is sufficient for accurate cytologic interpretation [15]. Yet, in a recent study [23] by Hasteh and Pang, they found that only one TP slide is representative of the specimen and is sufficient to make an accurate diagnosis. Irizar et al in their investigation reported that TP slides improve the diagnostic accuracy of thyroid FNAB samples. On the other hand, Biscotti et al found that TP slides offer similar diagnostic accuracy to that of conventional smear preparations [11].

A major advantage of CB preparation is the ability of performing special stains or immunocytochemical stains if needed [18]. This has been very helpful in FNAB of other organs especially the liver to differentiate metastatic tumors from primary liver carcinoma. However, there have been recent studies demonstrating the ability of performing immunocytochemical stains on TP slides, including those of the thyroid FNAB, with very good results [10,20,24,25]. A recent study by Rossi et al regarding immunocytochemical stains evaluation of thyroid neoplasms on TP slides from thyroid FNAB revealed that combining the cytomorphology with the immunocytochemical stain panel of HBME-1, Galectin-3 and RET was effective in distinguishing follicular lesions requiring surgery from those lesions requiring only follow-up [24]. However, the results obtained with this approach have not been directly compared with same specimens processed by traditional method on CB material.

Some authors have also elaborated on the cytologic features of specific thyroid lesions, such as follicular lesions and papillary carcinoma, on TP slides of thyroid FNAB specimens and described the differences from the features seen on traditional smears or CB slides [26,27].

In summary, we conclude that TP slide preparation in thyroid FNAB is more useful than CB slide preparation. Our study shows that the diagnostic yield of TP slides is at least twice that of CB slides in detecting thyroid lesions on FNAB, including atypical/neoplastic lesions, except for degenerative cysts. We further believe that TP slides should be made in addition to but not in lieu of direct (Diff-Quik and Papanicolaou stained) smears as they complement each other.

## List of abbreviations

FNAB: fine needle aspiration biopsy

TP: Thin Prep

CB: cell block

## Competing interests

The authors verify that they have no connection or interests to any company or product mentioned in this article.
